# Pharmacological activities and effective substances of the component-based Chinese medicine of *Ginkgo biloba* leaves based on serum pharmacochemistry, metabonomics and network pharmacology

**DOI:** 10.3389/fphar.2023.1151447

**Published:** 2023-03-10

**Authors:** Hongbao Liang, Jingchun Yao, Yu Miao, Ying Sun, Yanbing Gao, Chenghong Sun, Rui Li, He Xiao, Qun Feng, Guofei Qin, Xiaoyan Lu, Zhong Liu, Guimin Zhang, Feng Li, Mingguo Shao

**Affiliations:** ^1^ School of Pharmacy, Shandong University of Traditional Chinese Medicine, Jinan, China; ^2^ Center for Drug Safety Evaluation, Shandong New Time Pharmaceutical Co., Ltd., Linyi, China; ^3^ State Key Laboratory of Generic Manufacture Technology of Chinese Traditional Medicine, Lunan Pharmaceutical Group Co., Ltd., Linyi, China

**Keywords:** *Ginkgo biloba*, serum pharmacochemistry, metabonomics, network pharmacology, tyrosinase, hypertension

## Abstract

As a potential drug candidate for the treatment of hypertension and complications, it is speculated that the component-based Chinese medicine of *Ginkgo biloba* leaves (GBCCM) which mainly composed of flavonoid aglycones (FAs) and terpene lactones (TLs) may have different pharmacological effects at different doses or ratios. Taking the normal mice as the study object, metabonomics was conducted by giving different doses of GBCCM. Based on the components of GBCCM absorbed into the blood, the network pharmacological prediction was carried out. By integrating the results of metabonomics and network pharmacology, predict the possible pharmacological effects of GBCCM and conduct experimental verification. It was found that eight of the 19 compounds in GBCCM could be absorbed into the blood. GBCCM mainly affected the signal pathways of unsaturated fatty acid, pyruvate, bile acid, melanin and stem cells. It was speculated that GBCCM might have activities such as lowering blood pressure, regulating stem cell proliferation and melanogenesis. By establishing the models of mushroom tyrosinase, rat bone marrow mesenchymal stem cells (BMSCs) and spontaneously hypertensive rats (SHRs), we found that FAs and TLs showed synergistic effect in hypertension and tyrosinase models, and the optimal ratio was 3:2 (4.4 mg/kg) and 1:1 (0.4 mg/ml), respectively. As effective substances, FAs significantly promoted the proliferation of rat BMSCs on the third and fifth days at the concentration of 0.2 μg/ml (*p* < 0.05). GBCCM showed a variety of pharmacological effects at different doses and ratios, which provided an important reference for the druggability of GBCCM.

## 1 Introduction

GBE has a variety of pharmacological effects, such as vasodilation, blood lipid regulation, platelet activating factor antagonism, ischemic injury protection, anti-inflammatory and anti-tumor (Huang et al., 2013; Abdel-Zaher et al., 2017). *Ginkgo biloba* preparations with GBE as the main raw material are used in the treatment of cardiovascular and cerebrovascular diseases in clinical practice, such as coronary heart disease, cerebral infarction and memory loss, but there still exist some problems as follows: 1) Flavonoids and TLs are the main active components of GBE, but the total content of them only about 30% (Liu et al., 2021). Whether or not other components of GBE play a role in reducing toxicity and improving efficacy is unknown. 2) The activities of flavonoids in regard to preventing reperfusion injury, lowering blood lipid, enhancing memory and immune regulation are all based on their antioxidant effects (Crascì et al., 2018; Shen et al., 2022), and the antioxidant activity of FAs is stronger than flavonol glycosides (Odontuya et al., 2005). However, flavonoids of GBE mainly exist in form of the flavonol glycosides (Crespy et al., 1999). 3) The ratio between flavonoids and TLs of GBE is basically fixed, but it may not be the best.

Our research group prepared a component-based Chinese medicine of *Ginkgo biloba* leaves (GBCCM) mainly composed of flavonoid aglycones (FAs) and terpene lactones (TLs) from *Ginkgo biloba* leaves. FAs were obtained by converting flavonol glycosides into aglycones through hydrolysis *in vitro* and purification. However, TCM or natural products have the characteristics of multi-targets, multi-pathways and multi-pharmacological activities (Jiang et al., 2022; Zhao et al., 2022). GBCCM may exhibit a variety of pharmacological effects and complex dose-effect relationship at different drug concentrations and composition ratios. Therefore, solving the doubts is conducive to evaluating the potential value of GBCCM.

As a newly developed technology in recent years, metabonomics can be used to characterize the changes of endogenous substances in organisms caused by drug effects (Yang et al., 2005; Lyu et al., 2022). Although the drug may show different effects under physiological and pathological conditions, the potential action characteristics and pharmacological activities of the drug may be inferred from the analysis of the changes of differential metabolites and metabolic pathways at different doses under physiological conditions. Network pharmacology is a new cross discipline based on the theory of system biology, which uses bioinformatics and network analysis methods to analyze biological systems, study the mechanism of drug action from the system level, and carry out multi target drug molecular design (Zhou et al., 2020; Wang et al., 2021). It can help us find the complex action rules of traditional Chinese medicine by combing the relationship among components, targets and pathways.

In this study, integrating network pharmacology and serum metabolomics to reveal the relationship between blood components *in vivo*, drug concentration and signal pathways, so as to predict the possible pharmacological activities of GBCCM. The potential activities will be verified by using molecular, cellular and animal models. The optimal concentrations and composition ratios of GBCCM will also be explored. It is hoped that this study can provide data support for the clinical development of GBCCM.

## 2 Materials and methods

### 2.1 Chemicals

Tween-80 was purchased from Nanjing Well Pharmaceutical co., LTD. (Nanjing, China). Ethanol (AR grade), phosphate acid (AR grade), ethyl acetate (AR grade), hydrochloric acid (AR grade), n-hexane (AR grade) were all purchased from Sinopharm Chemical Reagent Co. LTD. (Shanghai, China). Standards of ginkgolide A ≥ 95%, ginkgolide B ≥ 95%, ginkgolide C ≥ 95%, ginkgolide J ≥ 95%, bilobalide ≥ 98%, quercetin ≥ 98%, isorhamnetin ≥ 98%, kaempferol ≥ 98% were purchased from National Institutes for Food and Drug Control (Beijing, China). Formic acid (MS grade), acetonitrile (HPLC grade), triffuoroacetic acid (HPLC grade), tetrahydrofuran (HPLC grade) and methanol (HPLC grade) were acquired from Merck (Darmstadt, Germany). Fetal Bovine Serum (FBS) was obtained from GIBCO (Australia Origin). DME/F12, PBS buffer, Trypsin, and Penicillin-streptomycin were purchased from Hyclone (Logan, Utah, United States). Anti-CD90-PerCP, anti-CD45-FITC, anti-CD29-PE and anti-CD34-Alexa Fluor 647 were acquired from BD Biosciences (San Diego, United States).

### 2.2 Animals

Mice (Weight: 18 ± 2 g), Wistar rats and SHRs (Weight: 200 ± 20 g) were purchased from Beijing Weitong Lihua Experimental Animal Technology Co., Ltd. (Beijing, China; animal license number: SCXK [Jing] 2016-0006). All experimental animal procedures were carried out according to the Guide and Use of Laboratory Animals and approved by the Ethics Committee for Experimental Animals at State Key Laboratory of Generic Manufacture Technology of Chinese Traditional Medicine (Approved on 13 November 2019; No. NH-IACUC-2019-38) for minimizing animal suffering.

### 2.3 Preparation of the component-based Chinese medicine of *Ginkgo biloba* leaves (GBCCM)

**FIGURE 1:**
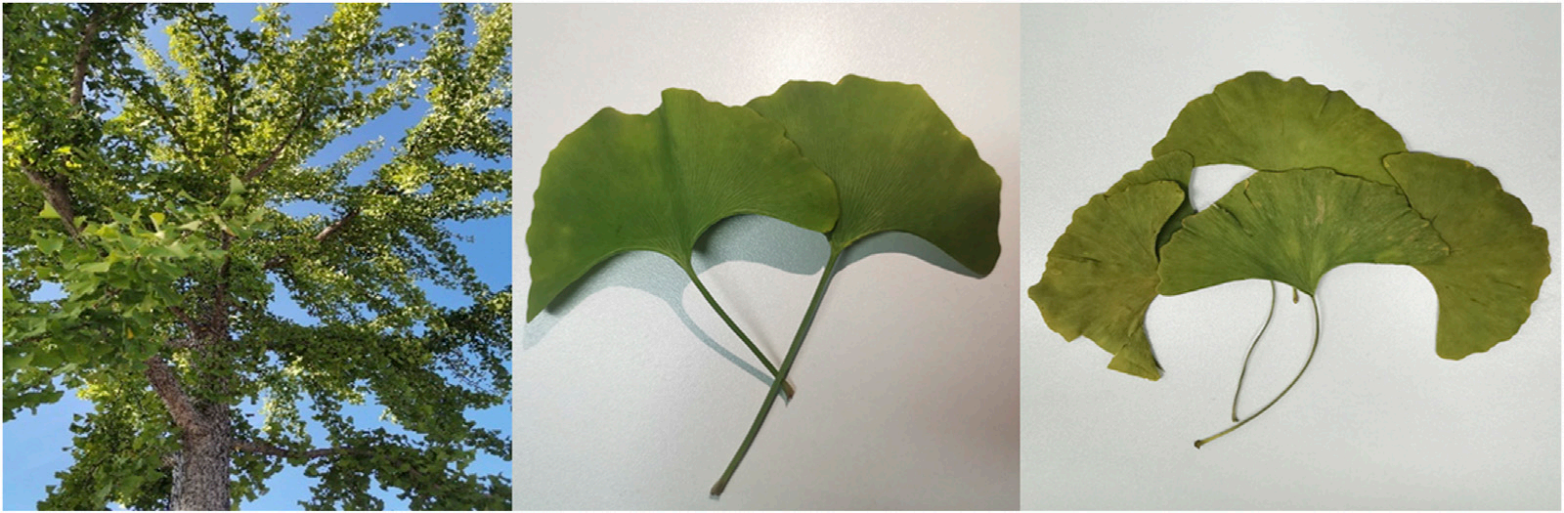
*Ginkgo biloba* plants (left), fresh leaves (middle), dried leaves (right).

*Ginkgo biloba* leaves were collected from Shandong, China in August 2021 and authenticated by the botanist Feng Li, Shandong University of Traditional Chinese Medicine, Shandong, China. The samples were dried, and stored without light (Figure 1). The preparation method for GBCCM was based on a previously published article (Liang et al., 2022b) with some modifications. GBCCM can be obtained by mixing FAs and TLs in different proportions as required. The specific method was as follows: *Ginkgo biloba* leaves (1 kg) were extracted two times with 50% ethanol, concentrated and ethanol precipitated. The supernatant was concentrated and purified with macroporous resin, and 50% of the eluent was collected. Then, the concentrated eluent was extracted twice with equal volume of ethyl acetate. Among them, flavonol glycosides were mainly enriched in the aqueous phase, and TLs were mainly enriched in the ethyl acetate phase. After secondary purification with macroporous resin, the aqueous phase was acid hydrolyzed at 80°C for 3.5 h. The hydrolysate was adjusted to neutral with NaOH and concentrated to dry. Performing reflux extraction with ethyl acetate, and concentrating the extract until to dry. FAs (2.8 g) would be obtained through crystallization in ethanol/water (1:3) system. The ethyl acetate phase was deacidified and decolorized twice with activated carbon. TLs (5.0 g) would be obtained through crystallization in ethanol/water (1:1.5) system.

### 2.4 Preparation of samples for analysis *in vivo*

Twenty-four mice were randomly divided into control group, GBCCM low-dose group and GBCCM high-dose group according to body weight with eight mice in each group. The mice in low-dose group and high-dose group were orally administered with GBCCM at the dose of 4.4 mg/kg and 44 mg/kg (FAs: TLs = 3: 2) respectively. The control group was given equal volume of 0.5% CMC-Na, once a day. Before the last administration, the mice were fasting for 12 h (Free drinking water). 30 min after drug supplementation on the day 5, all animals were anesthetized with ketamine (100 mg/kg, i.p.) and xylazine (7.5 mg/kg, i.m.).

Blood was collected from the abdominal inferior vena cava. After placing at room temperature for 30 min, the blood samples were centrifuged (Z326K, Hermle, Germany) at 3,500 rpm for 10 min. 1,000 μL methanol-acetonitrile (V: V = 2: 1) was added to 300 μL serum sample, and the mixture was agitated for 2 min using a vortex agitator followed by centrifugation at 18,000 rpm for 10 min. The supernatant was blown to dry with N_2_at 35°C and the residue was dissolved again in 200 μL methanol-water (V: V = 8: 2). Subsequently, the samples were centrifuged at 12,000 rpm for 15 min at 4°C. The supernatant was used for LC-MS/MS analysis. The QC samples were prepared by pooling the same volume of supernatant from each of the samples.

### 2.5 Serum pharmacochemistry

#### 2.5.1 Chromatographic and mass spectrometric conditions

The qualitative analysis of the serum samples from the control group and high-dose group was carried out by UPLC-Q-Exactive-MS/MS (Thermo Fisher Scientiffc, United States). The liquid chromatographic separation of all analytes was carried out on a Waters-ACQUITY UPLC HSS analytical column (2.1 × 100 mm, 1.8 µm) at 30°C with 0.1% formic acid-water as phase A and acetonitrile as phase B. The flow rate was maintained at 0.2 mL/min and the injection volume was 2 µL. The column temperature was 30°C. The gradient elution was as follows: 0 min, 15%B; 30 min, 40%B; 33 min, 55%B; 35 min, 15%B.

The Q-Exactive-Orbitrap-MS was coupled to the LC system *via* an electrospray ionization interface. Ultrahigh-purity helium (He) was used as collision gas and high-purity nitrogen (N_2_), as nebulizing gas. Mass spectrum was recorded in the range m/z 150-1,500 in the positive and negative ion modes. The conditions were as follows: ion spray voltage was 3.5 kV, capillary temperature was 350°C, collision voltage was 40 eV, the sheath and auxiliary gases flow rate were 35 and 10 (arbitrary units), respectively. The contents of GBCCM were analyzed by HPLC-CAD (Liang et al., 2022a).

#### 2.5.2 Data analysis

The chemical components of GBCCM were identified using the PeakView 2.1 software that was supplied with the instrument. The data of blank serum and GBCCM containing serum were imported into the software. Blank serum was taken as the normal control group, the information such as retention time, accurate molecular weight, accurate mass charge ratio and secondary ion fragments were compared with the *in vitro* components, and if they were consistent, they were identified as prototype components. Previous studies (Pietta et al., 1997; Li et al., 2012) had shown that flavonoids and terpene lactones of GBE are metabolized by the liver *via* phase II reactions after absorption into blood, and mainly forming glucuronic acid, sulfate or glutathione conjugates. The conjugated compounds were not found to have biological activity, so this study focused on the components absorbed into the blood rather than phase I and phase II metabolites.

### 2.6 Metabonomics study

#### 2.6.1 Chromatographic and mass spectrometric conditions

The liquid chromatographic separation of all analytes was carried out on a Waters-HSS T3 C18 analytical column (2.1 × 100 mm, 1.7 μm) with 0.01% formic acid-water as phase A and acetonitrile as phase B. The flow rate was maintained at 0.20 mL/min. The column temperature was 40°C and the injection volume was 2.0 μL. The gradient elution conditions were as follows: 0 min, 5%B; 2 min, 5%B; 4 min, 45%B; 23 min, 60%B; 27 min, 100%B; 32 min, 5%B; 34 min 5%B.

The UPLC-LTQ-Oribitrap-MS (Thermo Scientific, Santa Clara, United States) was coupled to the LC system *via* an electrospray ionization interface. Mass spectrum was recorded in the range m/z 50-1800 in positive and negative ion mode. The spray and capillary voltages were 4.0 kV and 35.0 V, respectively. The capillary temperature was 350°C and the tube lens voltage was set to 110V. N_2_ (purity > 99.99%) was used as both the sheath gas (40 arb) and auxiliary gas (20 arb). Data-dependent acquisition (ddms3) of high-resolution Fourier transform (TF, full scan, resolution 30,000) and CID fragmentation were used for positive and negative ion data acquisition.

#### 2.6.2 Data pre-processing and multivariate pattern recognition

The collected LC–MS/MS raw data files were imported into Compound Discoverer 3.1 software (Thermo, MA, United States) to obtain matched peak data. The parameters were set as follows: quality range, 100-1,500; quality deviation, 5 × 10^−6^; retention time deviation, 0.05 min; SNR threshold, 3. Peak area normalization. Normalized data were imported into SIMCA-P 13.0 software for principal component analysis (PCA), and orthogonal partial least squares discriminant analysis (OPLS-DA). A 200-iteration permutation test was used to verify the robustness of the supervised OPLS-DA model and to assess the degree of overfitting. The differential metabolites were selected on the basis of the combination of a statistically significant threshold of variable influence on projection (VIP) values obtained from the OPLS-DA model and *p* values from a two-tailed Student’s t-test on the normalized peak areas, where metabolites with VIP > 1.0 and *p* < 0.05 were considered as differential metabolites.

The HMDB database and the mass spectrometry ion fragments were used to identify the selected compounds. The positive and negative ion data were combined into a data matrix table containing all the information extracted from the original data and used for subsequent analysis. SPASS 23.0 software was used to conduct one-way analysis of variance (ANOVA) for differential metabolite data. Pathway enrichment analysis was performed on the Metaboanalyst platform (https://www.metaboanalyst.ca/).

### 2.7 Network pharmacology analysis

The molecular targets of the constituents absorbed into the blood were searched from STITCH 5.0 (http://stitch.embl.de/) and bioinformatics analysis tool for molecular mechanism of traditional Chinese medicine (BATMAN-TCM, http://bionet.ncpsb.org/batman-tcm/). After removing duplicates, the related targets of GBCCM were obtained. All the intersected targets were normalized to their official symbols by the UniProt data-base (https://www.uniprot.org/). The Kyoto Encyclopedia of Genes and Genomes (KEGG) pathway enrichment analyses were performed based on the online platform KOBAS (http://kobas.cbi.pku.edu.cn/index.php) with *p* < 0.01. The pathways were presented as bubble plots using the “pathview” package in the R software, and the “active ingredient-target” network was visualized using the Cytoscape software to visualize.

### 2.8 Tyrosinase inhibitory activity

#### 2.8.1 Inhibitory effect of GBCCM on tyrosinase activity *in vitro*

The tyrosinase inhibitory activity of FAs and TLs was evaluated detected according to the instruction of the reagent kit (Sigma-Aldrich, St. Louis, MO, United States). Weigh appropriate amounts of FAs, TLs and kojic acid to prepare a stock solution of 40 mg/mL with 20% tween-80 ethanol solution. Dilute them to the final concentration with ultrapure water before testing (Table 1). Adding 20 μL test samples and tyrosinase analysis buffer to the sample hole (S) and control hole (EC) to be tested respectively. Adding 50 μL tyrosinase solution, incubating at 25°C for 10 min, and then adding 30 μL tyrosinase substrate solution to each well, incubating at 25°C for 30–60 min. Two time points (T1 and T2) were selected within 30–60 min and a full wavelength microplate reader was used for acquiring absorbance values of AbT1 and AbT2 at 510 nm. The tyrosinase activity inhibition rate (A%) was calculated according to the following formula (1).

$$A\%=\left[\left(AbT2-AbT1\right)\;EC-\left(AbT2-AbT1\right)\;S\right]/\;\left(AbT2-AbT1\right)\;EC\times 100\%$$ (1)

**TABLE 1:** Final concentration of each sample to be tested.

	A (mg/ml)	B (mg/ml)	C (mg/ml)	D (mg/ml)	E (mg/ml)
FAs	0.002	0.02	0.2	0.5	1.0
TLs	0.002	0.02	0.2	0.5	1.0
Kojic acid	0.002	0.02	0.2	0.5	1.0
FAs + TLs	0.2 + 0 (2:0)	0.2 + 0.1 (2:1)	1.2 + 0.2 (2:2)	0.1 + 0.2 (1:2)	0 + 0.2 (0:2)

#### 2.8.2 Molecular docking

The molecular structure of mushroom tyrosinase (AbTYR; PDB code: 2Y9X) was used for the docking studies. The structures of the ligand compound were drawn by ChemDraw ultra and saved in mol format. After being imported into the software for pretreatment, flexible docking was carried out. DS visualizer 3.5 software was used for analysis. The LibDock score, the CDocker energy, the CDocker interaction energy and the hydrogen bond formation of the ligand-receptor complex were comprehensively considered so as to determine the final steady conformation.

### 2.9 Proliferative activity of mesenchymal stem cells

#### 2.9.1 Isolation, cultivation and passage of rat BMSCs

The healthy Wistar rats of 3–4 weeks were killed after cervical spondylectomy, and the femur and tibia were separated under sterile conditions. Wash the bone marrow cavity repeatedly with serum-free DME/F12 medium until white. Centrifuge the cleaning solution at 1,200 rpm for 5 min and discard the supernatant. The cells were resuspended with DME/F12 culture medium containing 10% FBS and inoculated into the culture bottle. The cells were cultured in a 37°C, 5% CO_2_ incubator. Culture medium was changed every 48 h. When the cells adherent growth density reached about 85%, they were digested with 0.25% trypsin. The cell morphology was observed with an inverted phase contrast microscope. When most of the cell body retracted and became round, stop the digestion. The cells were subcultured at a ratio of 1:2.

#### 2.9.2 Detection of surface markers of rat BMSCs by flow cytometry

The third generation BMSCs were collected and resuspended after digestion with 0.25% trypsin. The cells were resuspended with 50 μl of pre-cooled PBS buffer, and an appropriate amount antibodies and corresponding homotypic control antibodies were added respectively. After incubating at room temperature in the dark for 30 min, add 1 ml PBS and shake it well. Centrifuge for 5 min at 1,200 rpm and discard the supernatant. Add an appropriate amount of PBS buffer, mix well, and then being detected by flow cytometry (CytoFLEX, Beckman Coulter, United States).

#### 2.9.3 Effect of GBCCM on rat BMSCs

CCK-8 method was used to detect the effects of FAs and TLs on the proliferation of BMSCs (Kong and Chen, 2020). The experiment was divided into nine groups, including control group (C), FAs low-dose group (GFL), medium-dose group (GFM), high-dose group (GFH), TLs low-dose group (GLL), medium-dose group (GLM), high-dose group (GLH), FAs + TLs (1:1) low-dose group (GFLL), medium-dose group (GFLM) and high-dose group (GFLH). The drug concentrations were 0.02 μg/ml (L), 0.2 μg/ml (M), 2 μg/ml (H) respectively.

The third generation BMSCs were inoculated into 96-well plates at the concentration of 2 × 10^4^ cells/well. 100 μL of test samples under different concentrations (five wells in each concentration) were added. The culture was terminated on 1, 3, 5 days. After 100 μL of basic medium and 20 μL of CCK-8 solution were added to each well, 96-well plates were kept in the incubator at 37°C for 2 h. The microplate reader was used for acquiring absorbance values (OD) at 450 nm.

### 2.10 Effect of GBCCM on blood pressure

Wistar rats were used as the normal group consisting of 10 rats. SHRs were divided into six groups with 10 rats in each group, including model group, FAs + TLs (5:0), FAs + TLs (4:1), FAs + TLs (3:2), FAs + TLs (2:3), FAs + TLs (1:4) and FAs + TLs (0:5) groups, and amlodipine besylate group, respectively. The normal group and the model group were given water (containing 0.5% sodium carboxymethyl cellulose) by gavage. FAs + TLs (4.4 mg/kg) and amlodipine besylate (0.5 mg/kg) were suspended in 0.5% sodium carboxymethyl cellulose and orally administered to SHRs once daily for 60 days as administration groups.

Blood pressure was measured at day 0, and 60 after the start of treatment. The rats were allowed to rest for at least 15 min at 30°C before systolic blood pressure (SBP) was measured by a tail-cuff method (BP-2000, Visitech Systems, Inc., Apex, NC, United States). Blood pressure was measured four times for each rat, and the mean value was recorded.

### 2.11 Statistical analysis

Statistical analysis was performed using SPSS software (Version 23.0; IBM SPSS Statistics Inc., Chicago, IL, United States). The results were expressed as mean ± SD, and three repeats were performed for each experiment. One-way analysis of variance (ANOVA) was performed to evaluate the differences in mean values. Significant differences were verified by the Tukey-Kramer honestly significant difference test (*p* < 0.05).

## 3 Results

### 3.1 Identification of chemical constituents of GBCCM before and after blood transfusion

**FIGURE 2:**
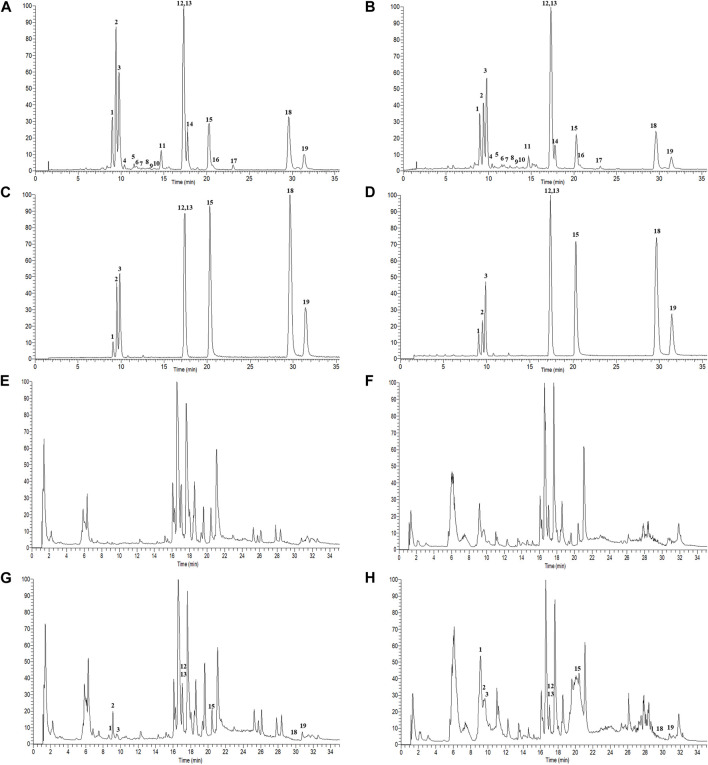
Total ion chromatograms of GBCCM before and after blood transfusion. Test solution (**(A)**, ESI-; **(B)**, ESI+), mixed standards (**(C)**, ESI-; **(D)**, ESI+), blank serum (**(E)**, ESI-; **(F)**, ESI+), medicated serum (**(G)**, ESI-; **(H)**, ESI+). The corresponding compounds of peaks 1-19 are shown in Supplementary Table S1.

A total of 19 components were identified from GBCCM by LC-MS/MS *in vitro* (Figure 2), including eight flavonoid glycosides, five flavonoid aglycones (FAs) and six terpene lactones (TLs) (Supplementary Table S1). FAs and TLs were the main components. The content of GBCCM (FAs + TLs) was reached 92.3%. Less flavonoid glycosides also had been identified, indicating that acid hydrolysis was incomplete.

**FIGURE 3:**
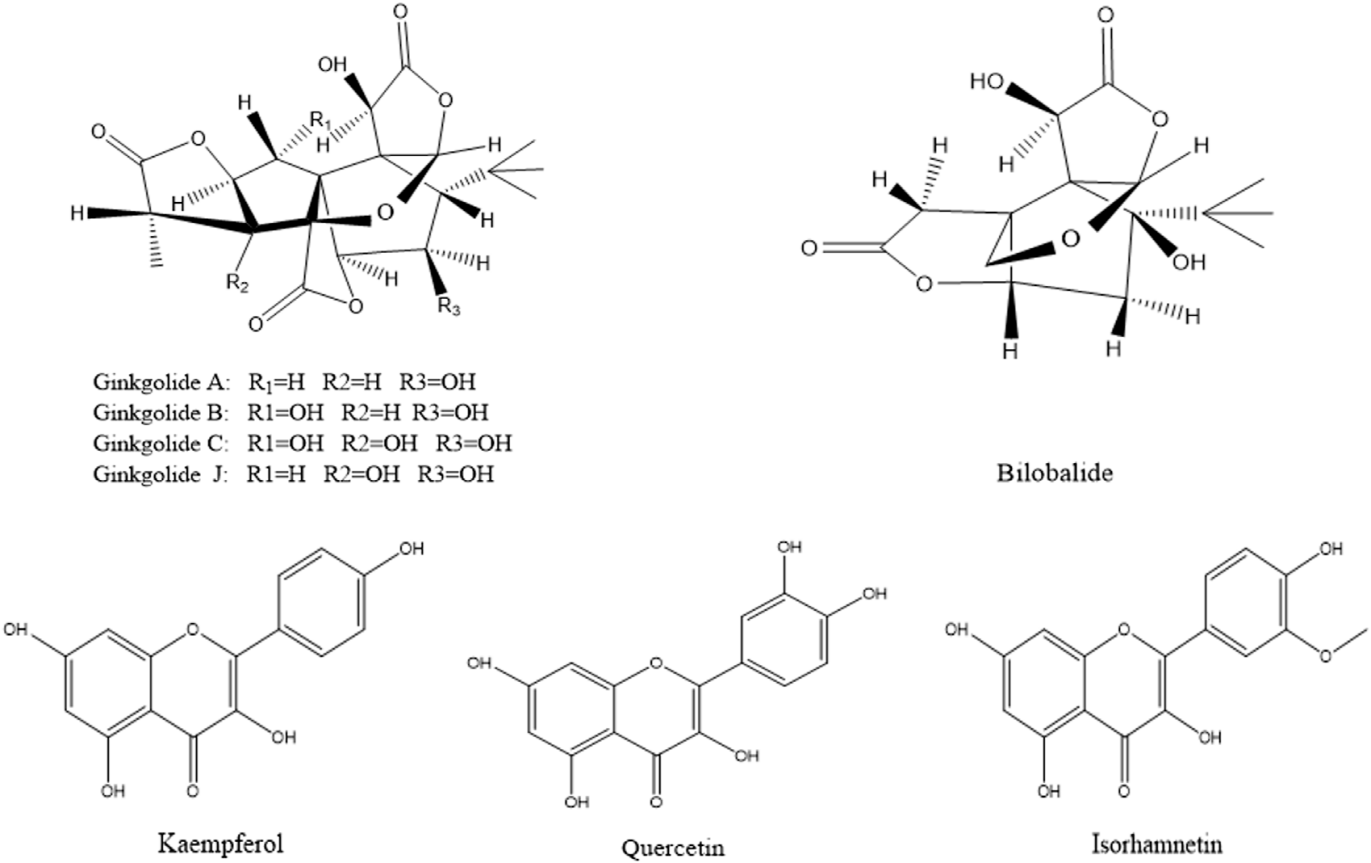
Chemical structures of components absorbed into the blood.

**FIGURE 4:**
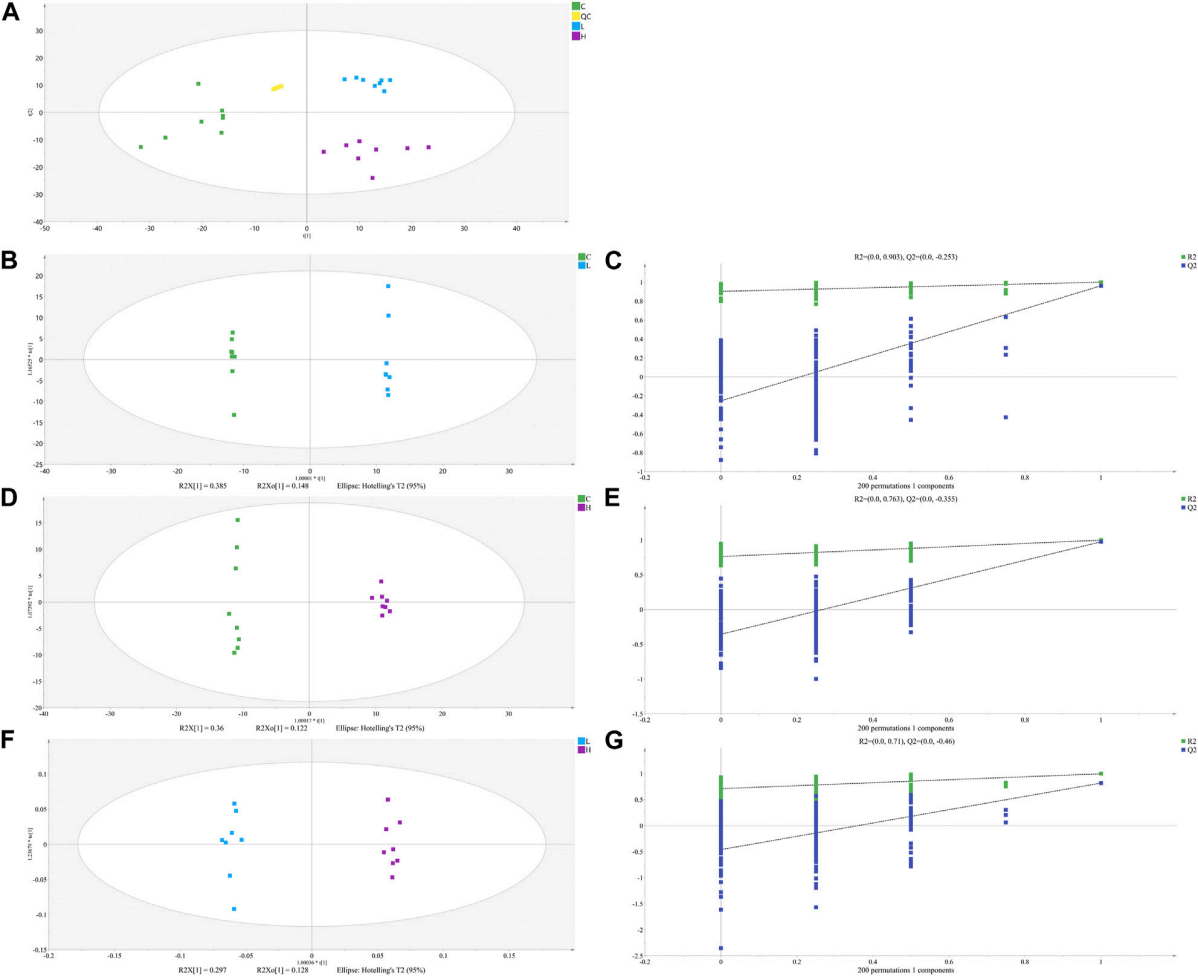
The PCA **(A)**, OPLS-DA **(B, D)** and **(F)** score plots of C, L and H and permutation test of OPLS-DA model **(C, E)** and **(G)**. **(C)** Control group samples, L: Low-dose group samples, H: High-dose group samples, QC: Quality control (QC) samples, R2: Goodness of fit, and Q2: Predictability of the models.

According to the method described in “2.5.1”, data were collected in both positive and negative ion modes. The total ion chromatograms (TICs) were shown in Figure 3. Using the analysis method described in “2.5.2”, a total of eight components absorbed into blood were identified, including three flavonoids (quercetin, kaempferol and isorhamnetin) and five terpene lactones (bilobalide, ginkgolide A, B, C and J). The structural formulas of these compounds were shown in Figure 4. Other compounds of GBCCM were not be identified, it may be because the concentrations of them were too low or they had been converted into phase I and phase II metabolites.

### 3.2 Metabonomics analysis

#### 3.2.1 UHPLC-MS/MS analysis of serum samples

The representative LC–MS/MS TICs of the serum samples were shown in Supplementary Figure S1. In the positive and negative ion mode, the chromatographic peaks of the control group are different from those of the low-dose group and high-dose group, indicating that there were obvious differences in endogenous metabolites among the three groups of serum samples.

#### 3.2.2 Statistical analysis of metabolic data by PCA and OPLS-DA

The unsupervised PCA is performed on all sample data to show theiroriginal classification status. The PCA scoring plots showed a tight aggregation of quality control (QC) samples, showing a good reproducibility of the instrument throughout the analysis period (Figure 4A). As expected, we observed a clear separation and clustering between the control group, low-dose group and high-dose group, with no extreme outliers to exclude, suggesting that there were significant differences at the metabolic level of mice. The supervised OPLS-DA was used to identify more specific metabolites among the groups. Such distributions and changing trends in aggregation and separation among control group, low-dose group and high-dose group became more apparent with OPLS-DA analysis (Figures 4B,D,F). To further verify the above observations, we performed a permutation procedure test using the OPLS-DA model with the same number of components. In total, 200 rounds of random permutations of the y variable were performed, and the results showed that while the R2 values (R2 represents the validity of the model and indicates the goodness of fit) were largely steady, the Q2 values which represents the accuracy of the model prediction were substantially decreased with increasing cycles of interaction validation (Figures 4C,E,G). We found that both goodness-of-fit parameters (R2 and Q2) calculated for the ranked data were lower than the corresponding original points on the right-hand side (1 on X-axis), and the intercepts of Q2 regression lines were all less than zero, indicating little overfitting in the original prediction model. Therefore, these analyses show that the separation model is statistically valid, and that the high value of predictability is not caused by overfitting. Therefore, we used these data for subsequent analyses.

#### 3.2.3 Identification and analysis of sifferential metabolites

**FIGURE 5:**
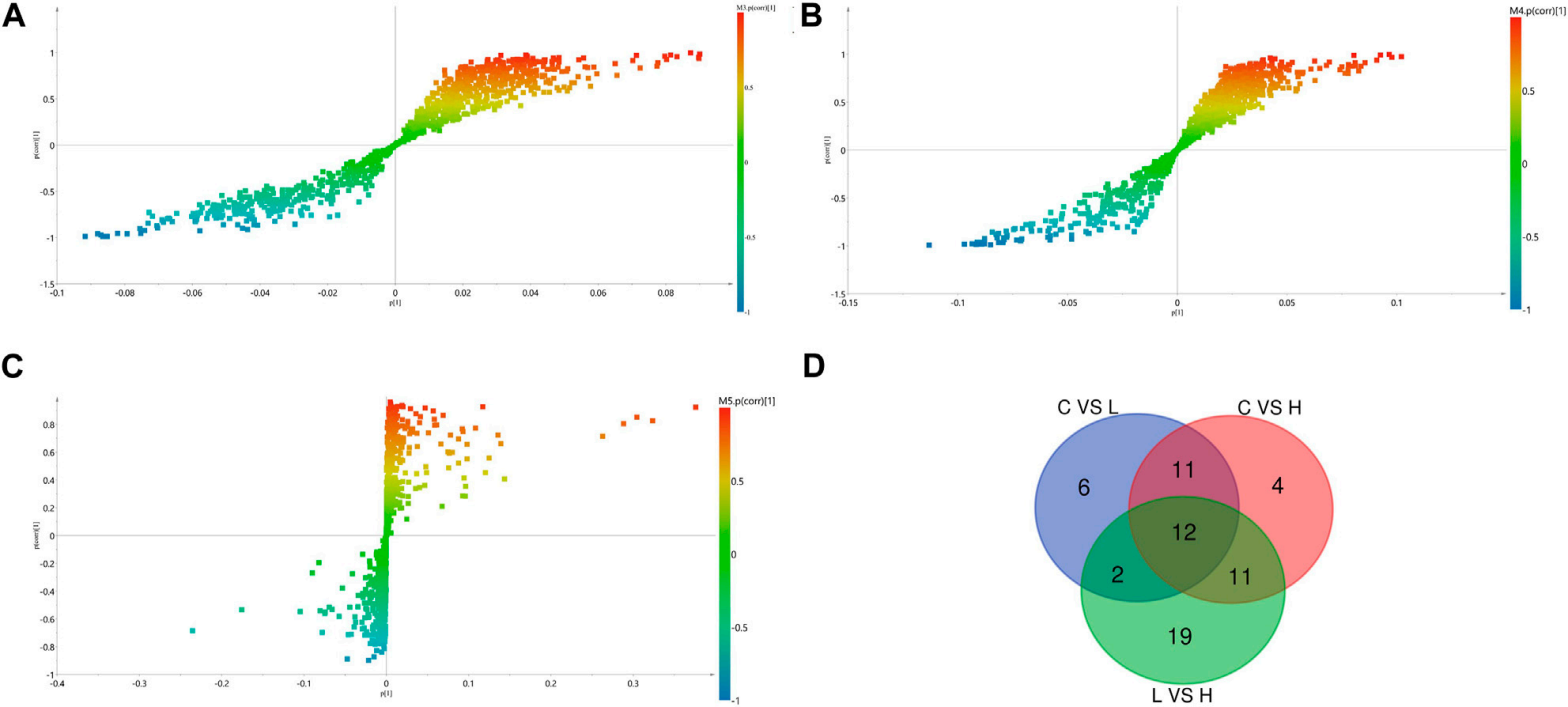
OPLS-DA S- Plot **(A, B)** and **(C)** and venn diagram of differential metabolites **(D)**. **(A)** Low-dose group vs. Control group, **(B)** High-dose group vs. Control group, **(C)** Low-dose group vs. High-dose group. **(C)** Control group, L: Low-dose group, H: High-dose group.

We identified the potential metabolites among the groups combined with S-plot obtained from OPLS-DA analysis. S-plot analysis (Figures 5A–C) represented the farther away metabolite ions from origin represent the higher VIP value of the ions, and the higher VIP value represents the greater contribute to the difference between the two sample groups. Based on VIP >1.0 in the OPLS-DA model and *p* < 0.05 in Student’s t-test, a total of 31 differential metabolites between the low-dose group and control group were identified, including 17 up-regulated and 14 down-regulated; A total of 38 differential metabolites between the high-dose group and control group were identified, including 29 up-regulated and nine down-regulated; And a total of 44 differential metabolites between the high-dose group and low-dose group were identified, including 20 up-regulated and 24 down-regulated. Through the venn diagram (Figure 5D), we found 12 common differential metabolites, which were linoleic acid, docosahexaenoic acid, palmitoleic acid, oleic acid, palmitic acid, myristic acid, stearic acid, 3,4-dihydroxyhydrocinnamic acid, 3-(3,4-dihydroxy-5-methoxy)-2-propenoic acid, 15H-11,12-EETA, l-lactic acid and 2,3-dihydro-2-S-glutathionyl-3-hydroxy bromobenzene. It was speculated that GBCCM might mainly affect unsaturated fatty acids and fatty acid related metabolic pathways in a dose-dependent manner.

#### 3.2.4 Enrichment analysis of serum metabolite pathway

**FIGURE 6:**
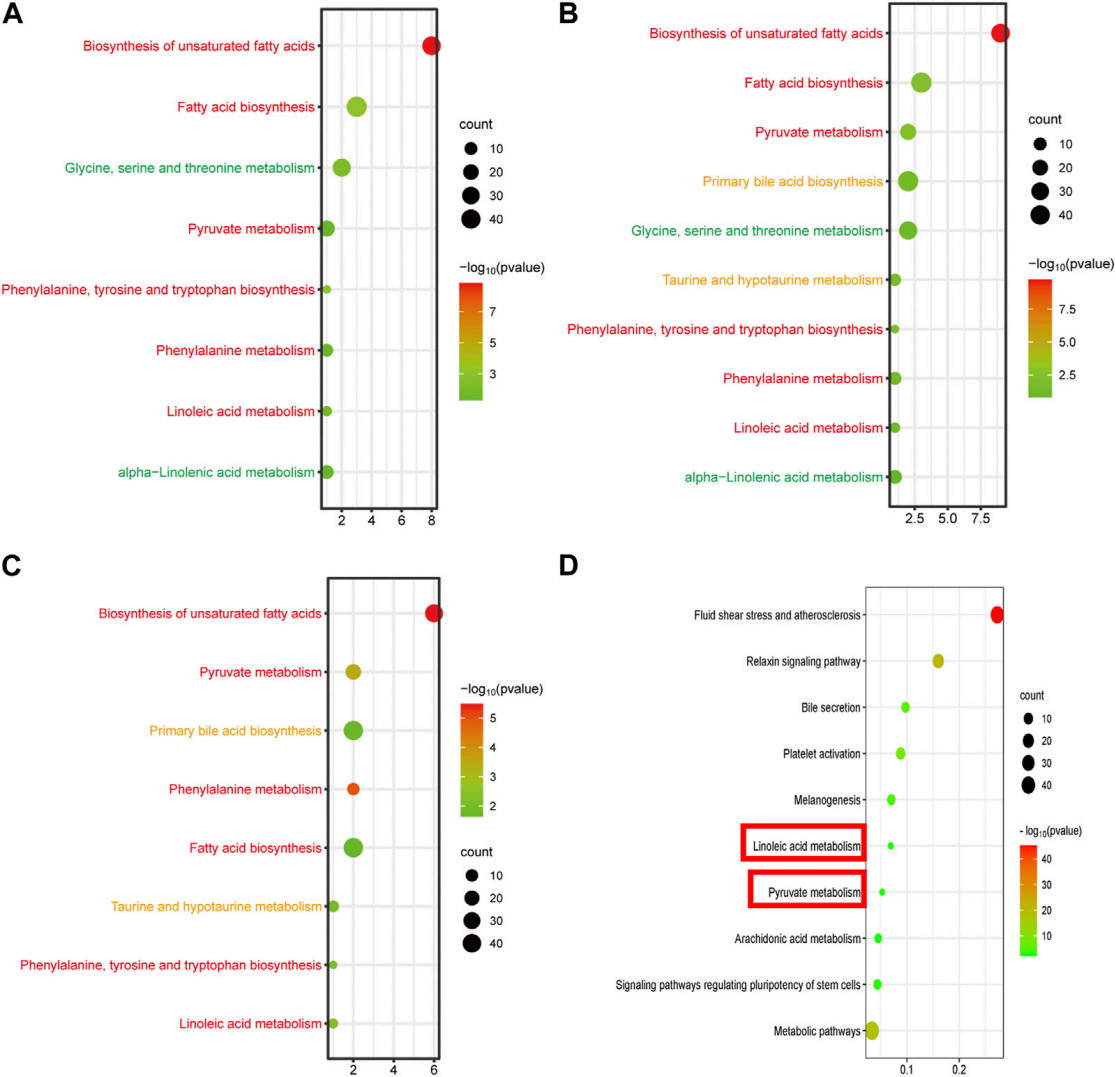
Bubble diagram of KEGG metabolic pathway enrichment analysis of significantly different metabolites **(A, B)** and **(C)**, and signal pathway enrichment analysis of components absorbed into the blood **(D)**. **(A)** Low-dose group vs. Control group, **(B)** High-dose group vs. Control group, **(C)** High-dose group vs. Low-dose group. The horizontal axis represents the rich factor, and the vertical axis represents the pathways. The bubble size represents the number of targets in the pathway. The bubble color indicates the magnitude of the -log_10_(p) values.

By setting adjusted *p* < 0.01 as the screening criteria, the KEGG pathway analysis of differentially abundant metabolites was performed by MetaboAnalyst 4.0 to identify the disturbed metabolic pathways caused by GBCCM. 10, eight and eight metabolic pathways were enriched among C VS H, C VS L, and L VS H, respectively (Figure 6). It can be seen from the above that at different doses, the active ingredients of GBCCM may affect the same or different metabolic pathways, showing the characteristics of multi-component and multi target effects. We found the biosynthesis of unsaturated fatty acids, fatty acid biosynthesis, pyruvate metabolish, phenylalanine, tyrosine and tryptophan biosynthesis, phenylalanine metabolish and linoleic acid metabolish were common metabolic pathways of the three groups. It indicated that the effective components absorbed into blood of GBCCM could affect these metabolic pathways in a dose dependent manner. Changes of metabolites of these common metabolic pathways have a strong correlation with cardiovascular and cerebrovascular diseases, cardiac hypertrophy, biological characteristics of mesenchymal stem cells (Smith et al., 2012; Abdelhamid et al., 2018; Borges et al., 2022), which also suggests that GBCCM may have the potential to treat hypertension, hyperlipidemia, and promote mesenchymal stem cell differentiation and proliferation.

### 3.3 Network pharmacology analysis

According to the prediction of the two online platforms, 303 targets of eight blood components were obtained and a total of 72 pathways were enriched through the online software KOBAS (*p* < 0.01). After removing irrelevant signal paths, the KEGG analysis showed that the target genes corresponding to the constituents absorbed into the blood were enriched in the signaling pathways related to linoleic acid metabolism, pyruvate metabolism, arachidonic acid metabolism, bile secretion, regulating pluripotency of stem cells and melanogenesis (Figure 6). Such a high consistency validated the accuracy of pathway analysis in the metabolomics. As an unsaturated fatty acid, Linoleic acid can prevent or reduce the occurrence of cardiovascular and cerebrovascular diseases, especially hypertension, hyperlipidemia, angina pectoris, coronary heart disease, arteriosclerosis and elderly obesity (den Hartigh, 2019). Pyruvate metabolism was closely related to myocardial hypertrophy (Cluntun et al., 2021)**.** Integrating the results of network pharmacology and metabonomics, we speculated that GBCCM might have the activities of regulating blood pressure, blood lipid, proliferation of stem cells and inhibiting melanin synthesis.

### 3.4 Tyrosinase inhibitory activity

#### 3.4.1 Inhibitory effect of GBCCM on tyrosinase activity *in vitro*

**FIGURE 7:**
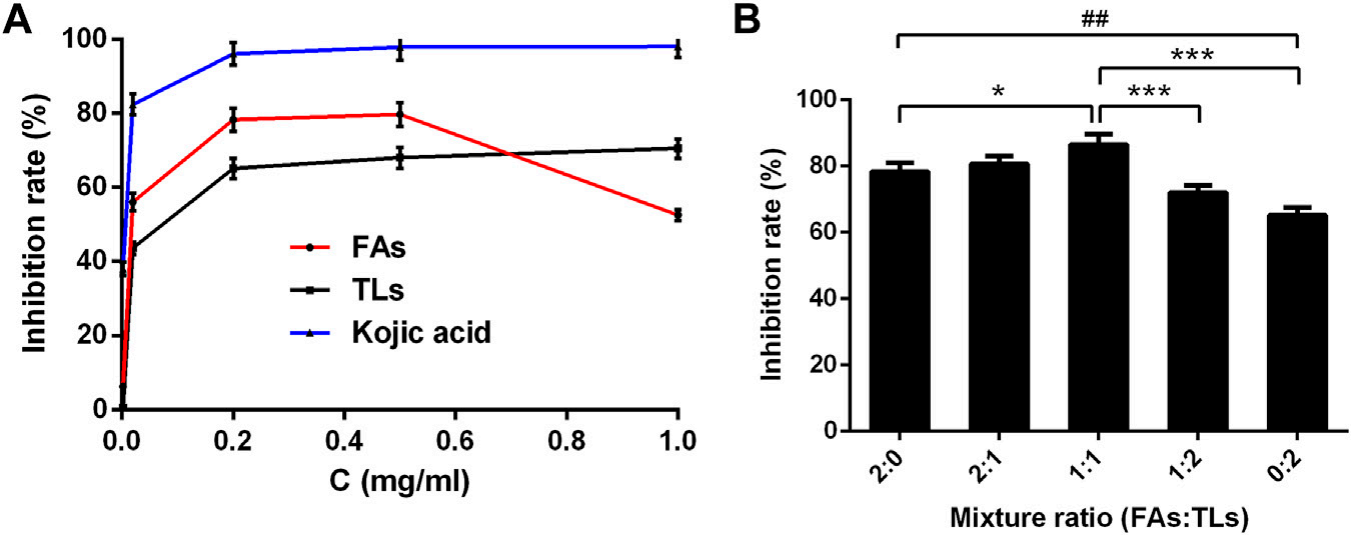
Tyrosinase inhibitory activity of FAs and TLs at different concentrations **(A)** and ratios **(B)**. *p < 0.05, ***p < 0.001 vs. FAs + TLs (1:1) group, ^##^ *p* < 0.01, vs. FAs + TLs (0:2) group.

As shown in Figure 7A, FAs and TLs exhibited potent inhibitory activities on tyrosinase dose-dependently with IC50 values of 0.02 ± 0.01 and 0.05 ± 0.01 mg/ml, respectively. Both of them were higher than that of kojic acid (0.006 ± 0.001 mg/ml).

The inhibition rates of FAs and TLs increased significantly with the increase of concentration in the range of 0–0.2 mg/ml. When the concentration reached 0.5 mg/ml, the tyrosinase inhibition rates of FAs and TLs reached 79.71% and 68.00% respectively. It could be seen from Figure 7B, FAs had stronger tyrosinase inhibitory activity than TLs at the same concentration (*p* < 0.01). When FAs and TLs were used together at a ratio of 1:1, the inhibition rate reached 86.55%, which was higher than the inhibition rate when used alone (*p* < 0.05, *p* < 0.001).

#### 3.4.2 The result of molecular docking

**FIGURE 8:**
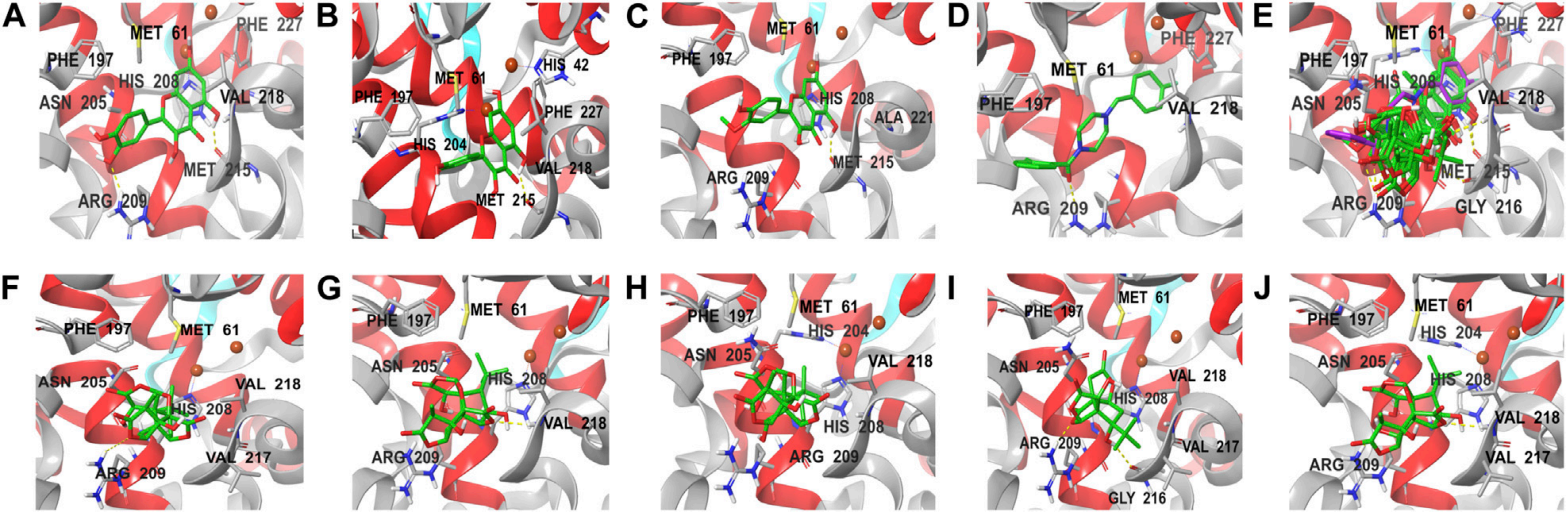
Molecular docking diagram of active components and key targets. The compound is presented in the form of stick, the mushroom tyrosinase is presented in the form of ribbon, and the yellow dotted line represents hydrogen bonding. **(A–J)** represent the docking of quercetin, kaempferol, isorhamnetin, original ligand, all compounds, ginkgolide A, ginkgolide J, ginkgolide B, bilobalide, ginkgolide C with mushroom tyrosinase, respectively.

The docking results of eight active ingredients and mushroom tyrosinase protein active sites were shown in Figure 8 **.** It was generally believed that the higher the docking score, the stronger the binding force between the compound and the target, the more stable the conformation and the stronger the potential role (Liang et al., 2021). Molecular docking results showed that the docking scores of the quercetin, kaempferol and isorhamnetin were close to or higher than those of the original ligand, indicating that these three components might have similar effects with the original ligand (Figures 8A–E). It could be seen from the molecular docking diagram that the flavone aglycone occupied the active center near two copper ions in tyrosinase. Through the hydrogen bond and hydrophobic force formed with multiple sites of the receptor, the affinity with the target protein was enhanced, and the stability of the conformation was also improved.

Although the docking scores of bilobalide, ginkgolide A, B, C and J were lower than those of the original ligands, the conformations match the pockets well, and they could also form hydrogen bonds and hydrophobic forces (Figures 8F,G). The collision fraction and polarity were within a reasonable range, indicating that they also had a certain tyrosinase inhibitory effect. The results of molecular docking were basically consistent with those of activity detection, which further proved the accuracy of docking results.

Molecular docking results showed that FAs could form more hydrogen bonds and hydrophobicity near the active center of tyrosinase than TLs due to their multiple phenolic hydroxyl groups, which also indicated that FAs may have stronger inhibitory activity than TLs. Among the three flavonoid aglycones, quercetin had the highest docking score, suggesting that its activity may be the strongest. This was mainly due to the fact that quercetin had the most phenolic hydroxyl groups on its C-ring and the strongest ability to chelate copper from the tyrosinase active center (Fan et al., 2017; Roulier et al., 2020).

### 3.5 Effects of GBCCM on the proliferation of rat BMSCs

Flow cytometry was used to detect the third generation BMSCs. As seen in Supplementary Figure S2, the positive expression of CD29 and CD90 was detected while CD45 and CD34 were negative, indicating that the BMSCs were successfully isolated.

**FIGURE 9:**
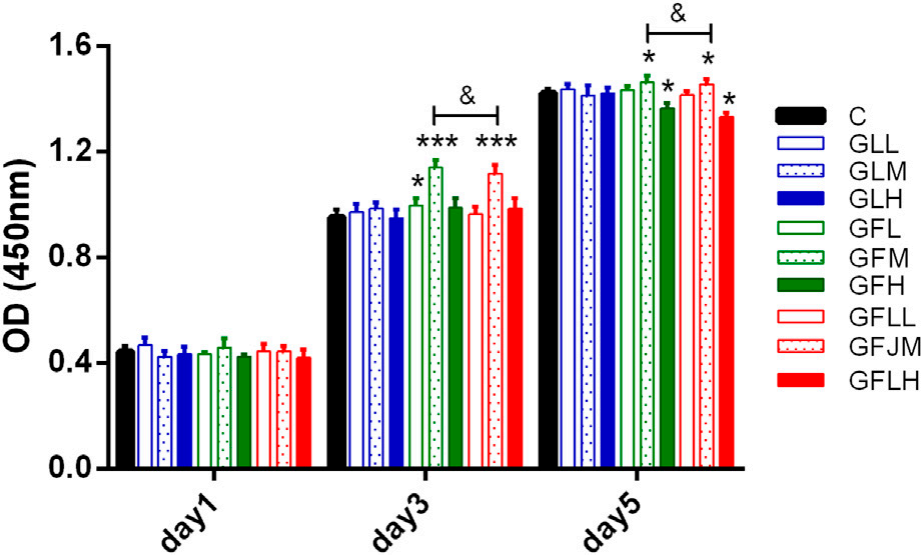
The effects of FAs and TLs on proliferation of rat BMSCs. *p < 0.05, ***p < 0.001 vs. Control group.

The effects of FAs and TLs on proliferation of BMSCs were shown in Figure 9. Compared with the normal control group, FAs significantly promoted BMSCs proliferation on the third and fifth days (*p* < 0.05, *p* < 0.001) at the concentration of 0.2 μg/ml. In each dose group, TLs had no effect on the proliferation of BMSCs (*p* > 0.05). When FAs and TLs (0.2 μg/ml) was used together at a ratio of 1:1, the effect was basically the same with FAs (*p* > 0.05), indicating that there was no synergistic effect between them. It was reported (Wu et al., 2016) that GBE did not show the activity of promoting the proliferation of rat BMSCs. It was speculated that the main reason may be that the content of free FAs in GBE was very little, and it was difficult to reach the effective concentration.

### 3.6 Effects of GBCCM on blood pressure

Previous study (Liang et al., 2022b) found that FAs and TLs had better antihypertensive activity at 4.4 mg/kg (3:2), but the proportion was not necessarily the best. Therefore, under the condition that the total dose remained unchanged, different ratio of FAs and TLs were designed based on the method of increasing proportion to study the optimal proportion.

**FIGURE 10:**
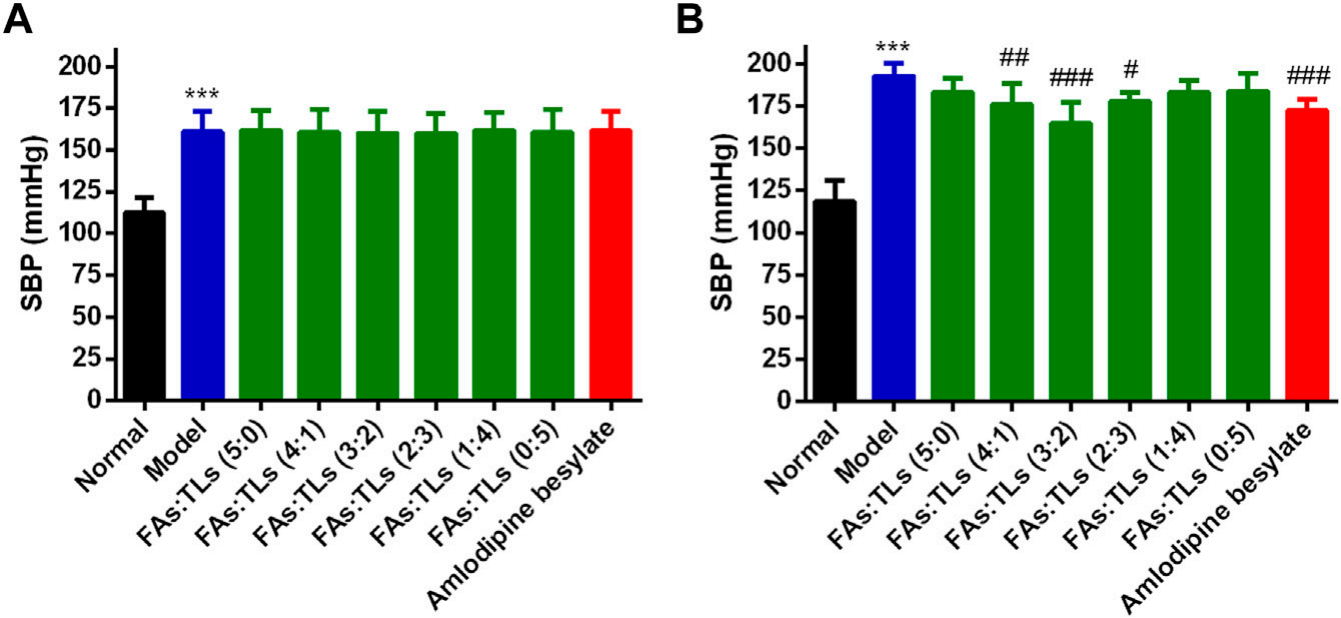
Effects on SBP of each group after repeated administration for 0 days **(A)** and 60 days **(B)**. ***p < 0.001 vs. Normal group, ^#^ *p* < 0.05, ^##^ *p* < 0.01, ^###^ *p* < 0.001 vs. Model group.

The therapeutic effect of each group on SHRs was shown in Figure 10. The mean SBP of model group was significantly higher than that of normal group (*p* < 0.01) (Figure 10A). After 60 days of treatment, FAs + TLs (5:0) group, FAs + TLs (1:4) group and FAs + TLs (0:5) group had no significant improvement on SBP (*p* > 0.05), while FAs + TLs (4:1) group (*p* < 0.01), FAs + TLs (3:2) group (*p* < 0.001) and FAs + TLs (2:3) group (*p* < 0.05) significantly improved the SBP of SHRs (Figure 10B). FAs + TLs (3:2) group and amlodipine besylate group showed significant hypotensive effect (*p* < 0.001), indicating that 3:2 was the optimal ratio between FAs and TLs.

## 4 Discussion

Many researches proved that the network pharmacology integrated with metabolomics strategy was effective method and can be used to study the effect and mechanism of TCM (Hua et al., 2019; Li et al., 2021). In this study, we innovatively predicted the possible pharmacological effects of GBCCM by integrating serum pharmacochemistry, network pharmacology and metabolomics. The potential activities were verified through experiments.

In metabonomics research, we found that GBCCM regulates six metabolic pathways including biosynthesis of unsaturated fatty acids, fatty acid biosynthesis, pyruvate metabolish, phenylalanine, tyrosine and tryptophan biosynthesis, phenylalanine metabolish and linoleic acid metabolish in a dose-dependent manner at two doses (low and high), which showed the characteristics of multi-components, multi-targets and multi-pathways of GBCCM. Changes of metabolites of these common metabolic pathways have a strong correlation with cardiovascular and cerebrovascular diseases, cardiac hypertrophy, biological characteristics of mesenchymal stem cells (Smith et al., 2012; Abdelhamid et al., 2018; Borges et al., 2022). Combined with the primary excimer ion peak and secondary fragment information provided by high resolution mass spectrometry, nineteen components, including eight flavonoid glycosides, five flavonoid aglycones (FAs) and six terpene lactones (TLs) were identified *in vitro* by referring to relevant literatures and comparing with reference standards. As the main active components, the content of FAs and TLs in GBCCM reached 92.3%. Compared with GBE, the chemical composition of GBCCM was relatively clear. According to pharmaceutical theory, the premise for the efficacy of traditional Chinese medicine is that the ingredients are absorbed into the blood (Yan et al., 2015). Therefore, it is particularly important to investigate the components of GBCCM that are really absorbed into the blood through serum pharmacochemistry. Only three FAs and five TLs of GBCCM were identified as the main blood components by LC-MS/MS. Other ingredients were not be identified, it may be because the concentrations of them were too low or they had been converted into phase I and phase II metabolites. Network pharmacological analysis showed that a total of 72 signal pathways were enriched, mainly involving linoleic acid metabolism, pyruvate metabolism, arachidonic acid metabolism, bile secretion, regulating pluripotency of stem cells and melanogenesis. Integrating the results of network pharmacology and metabonomics, we speculated that GBCCM might have the activities of regulating blood pressure, blood lipid, proliferation of stem cells and inhibiting melanin synthesis.

Tyrosinase is a copper containing polyphenol oxidase, which is the key enzyme involved in melanogenesis. The occurrence and treatment of pigment disorders, malignant melanoma, albinism and senile dementia are directly related to tyrosinase (El-Nashar et al., 2021). It has been reported (Xue et al., 2011; Solimine et al., 2016; Klomsakul et al., 2022) that flavonoid aglycones (FAs) in *Ginkgo biloba* extract (GBE) have certain tyrosinase inhibitory activity. In the tyrosinase inhibitory activity experiment, we found that FAs had a good inhibitory effect on tyrosinase, which was consistent with the literature reports. At the same time, TLs were also found to have significant inhibitory activity for the first time. The inhibitory activity of FAs showed a good dose dependence at low concentrations, and the inhibition rate was about 79.71% at 0.5 mg/ml. However, when the concentration continued to increase to 1.0 mg/ml, the inhibition rate decreased. The reason might be that FAs were easy to precipitate at high concentration due to their poor solubility. When FAs and TLs of GBCCM were used at a ratio of 1:1, the inhibition rate reached 86.55%, which was higher than the inhibition rate when used alone, indicating that they played a synergistic role when used together. Through molecular docking, it was speculated that the active components might play a role in the tyrosinase inhibitory through competitively bind to the active site in the enzyme with the substrate.

Bone marrow mesenchymal stem cells (BMSCs) are a kind of multipotent stem cells derived from bone marrow. They can not only provide hematopoietic support, but also have the potential of self-renewal, high proliferation and multi-directional differentiation (Liang et al., 2019). In recent years, many TCM extracts have been found to have certain effects on promoting the proliferation of BMSCs. Treatment of BMSCs with curcumin after 48 h, increased cell survival and proliferation in a dose-dependent manner (Attari et al., 2015). It was also found that Polygonatum, Plastrum testudinis, Panax notoginseng could promote the proliferation of stem cells (Li et al., 2011; Zong et al., 2015; Shen et al., 2018). GBE can promote the osteogenic differentiation of BMSCs, but had no significant effect on their proliferation (Wu et al., 2016). Through co-culture of BMSCs with GBCCM of different concentrations and proportions, we found that only FAs had a certain activity of promoting BMSCs proliferation at the concentration of 0.4 mg/ml. However, the activity was not strong enough to be further developed.

Hypertension is a chronic cardiovascular disease characterized by the rise of systemic arterial pressure, which can cause damage to heart, brain, kidney and other organs (Boesen and Kakalij, 2021). With the accelerated aging of the population, the prevalence of hypertension and its related diseases is increasing. Hypertension prevention and treatment has become a severe challenge facing the world’s public health (Frohlich, 2017). In recent years, the clinical application of western medicine combined with *Ginkgo biloba* preparation in the treatment of hypertensive patients shows that the antihypertensive effect of these two drugs is better than that of antihypertensive drugs taken alone (Mansour et al., 2011), but the antihypertensive active components and mechanism of GBE are still unclear. Our research group found that FAs and TLs mixed in a certain ratio had a better effect on lowering blood pressure when used alone (Liang et al., 2022b), but the composition ratios need to be further optimized. In this study, we found that the activity of FAs and TLs was not strong when used alone (*p* > 0.05) in the spontaneously hypertensive rat model. When the ratio of FAs to TLs was 3:2, GBCCM showed the best antihypertensive effect (*p* < 0.001), and there was no significant difference compared with amlodipine besylate, indicating that it had the potential clinical value.

**FIGURE 11:**
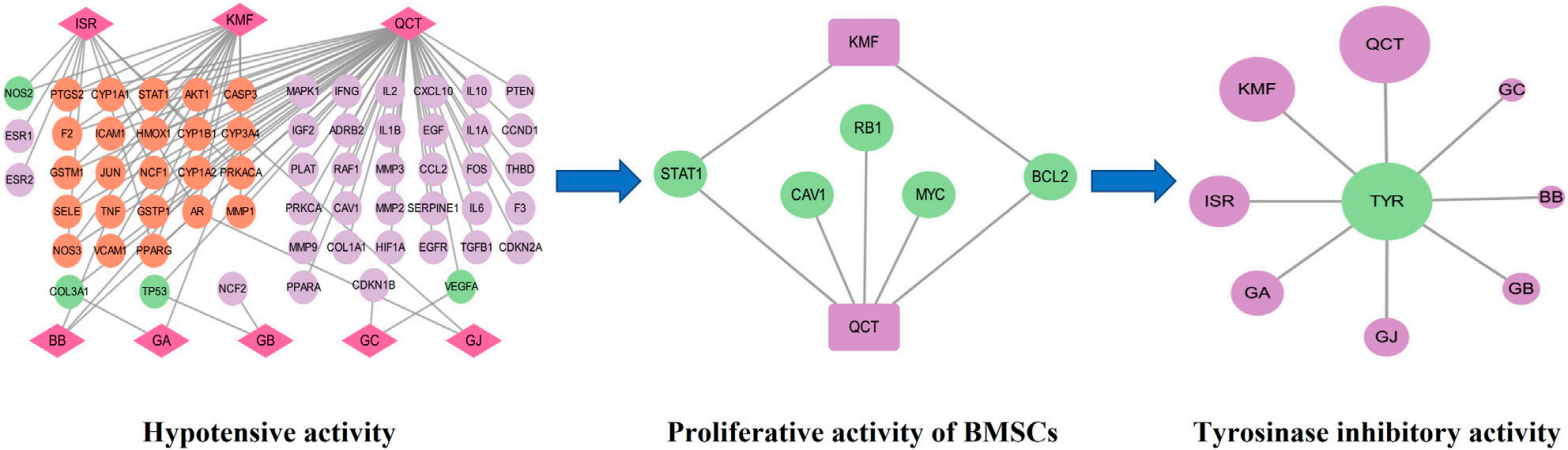
Network diagram of components-targets-pharmacological activities. QCT: Quercetin, KMF: Kaempferol, ISR: Isorhamnetin, BB: Bilobalide, GA: Ginkgolide A, GB: Ginkgolide B, GC: Ginkgolide C, GJ: Ginkgolide J, TYR: Tyrosinase.

Based on the above experiments, we found that GBCCM had significant tyrosinase inhibitory activity and blood pressure lowering activity, and there were complex network connections among components, targets, and pharmacological activities (Figure 11). Compared with GBE, GBCCM had the advantages of basically clear effective substances, relatively clear mechanism of action and convenience of prescription adjustment. We predict that GBCCM may become a new raw material for *Ginkgo biloba* preparations in the future. Strengthen the research on prescription ratio, dose-effect relationship and mechanism of TCM will help to improve the quality control level, enhance the efficacy and reduce the toxic and side effects, and it will also be the key point and breakthrough in the research of the modernization of TCM (Wang et al., 2016; Chen et al., 2019). As a novel mode for exploring the pharmacological activity of component-based Chinese medicine, we hope this study can bring some inspiration to other researchers.

## 5 Conclusion

In this study, the serum differential metabolites of mice after intragastric administration of different GBCCM doses were investigated, and the metabolic pathway enrichment was analyzed. Based on the components absorbed into the blood, the action network of active components in GBCCM was predicted by network pharmacology technology, and the enrichment analysis of signal pathway was also carried out. By integrating serum pharmacochemistry, metabonomics and network pharmacology, it was found that GBCCM mainly affected the signal pathways of unsaturated fatty acid, pyruvate, bile acid, melanin and stem cells. It was speculated that GBCCM might have activities such as lowering blood pressure, regulating stem cell proliferation and melanogenesis. The pharmacological activities of GBCCM were verified by molecular, cellular and animal models, and the effective substances of GBCCM in different models were also confirmed.

## Data Availability

The original contributions presented in the study are included in the article/Supplementary Materials, further inquiries can be directed to the corresponding authors.

## Supplementary material

Supplementary Figure S1 TICs of serum samples in positive and negative ion mode. Blank serum (**(A)**, ESI−; **(B)**, ESI+), low-dose serum (**(C)**, ESI−; **(D)**, ESI+), high-dose serum (**(E)**, ESI−; **(F)**, ESI+).

Supplementary Figure S2 Identification of rat BMSCs by flow cytometry.

Supplementary Table S1 The detected ion chromatogram of constituents in GBCCM.

## Abbreviations

TCM, Traditional Chinese medicine; GBCCM, Component-based Chinese medicine of *Ginkgo biloba* leaves; BMSCs, Bone marrow mesenchymal stem cells; SHRs, Spontaneously hypertensive rats; GBE, *Ginkgo biloba* extract; FAs, Flavonoid aglycones; TLs, Terpene lactones; PCA, Principal component analysis; OPLS-DA, Orthogonal partial least squares discriminant analysis; VIP, Variable influence on projection.
