# Supplementary figures and images for: Pharmacological activities and effective substances of the component-based Chinese medicine of Ginkgo biloba leaves based on serum pharmacochemistry, metabonomics and network pharmacology
Source: Front Pharmacol. 2023 Mar 10;14:1151447. doi: 10.3389/fphar.2023.1151447 (PMC10036596; doi:10.3389/fphar.2023.1151447)

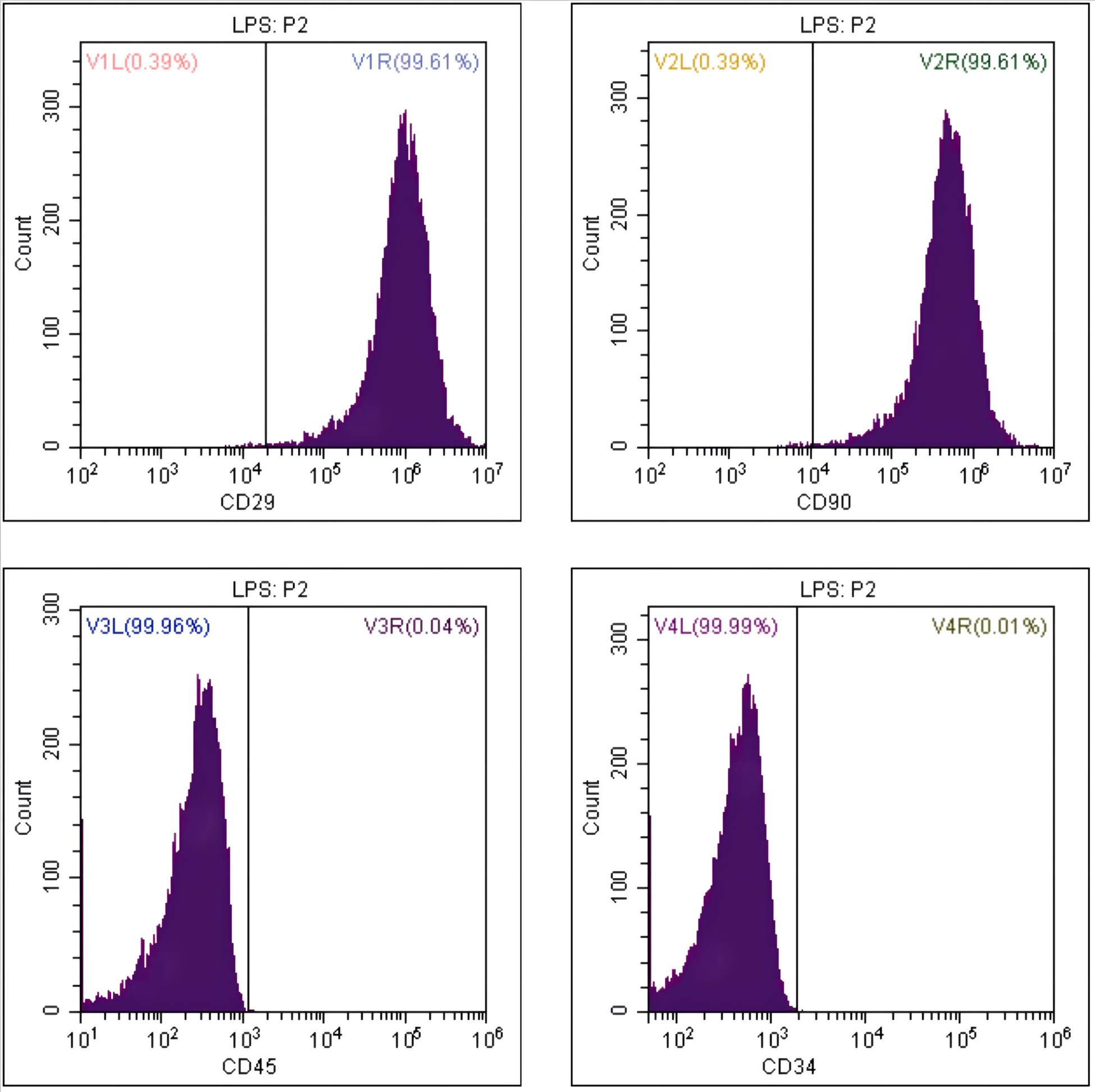

Supplement: Supplementary file 1 [file Image2.TIF]

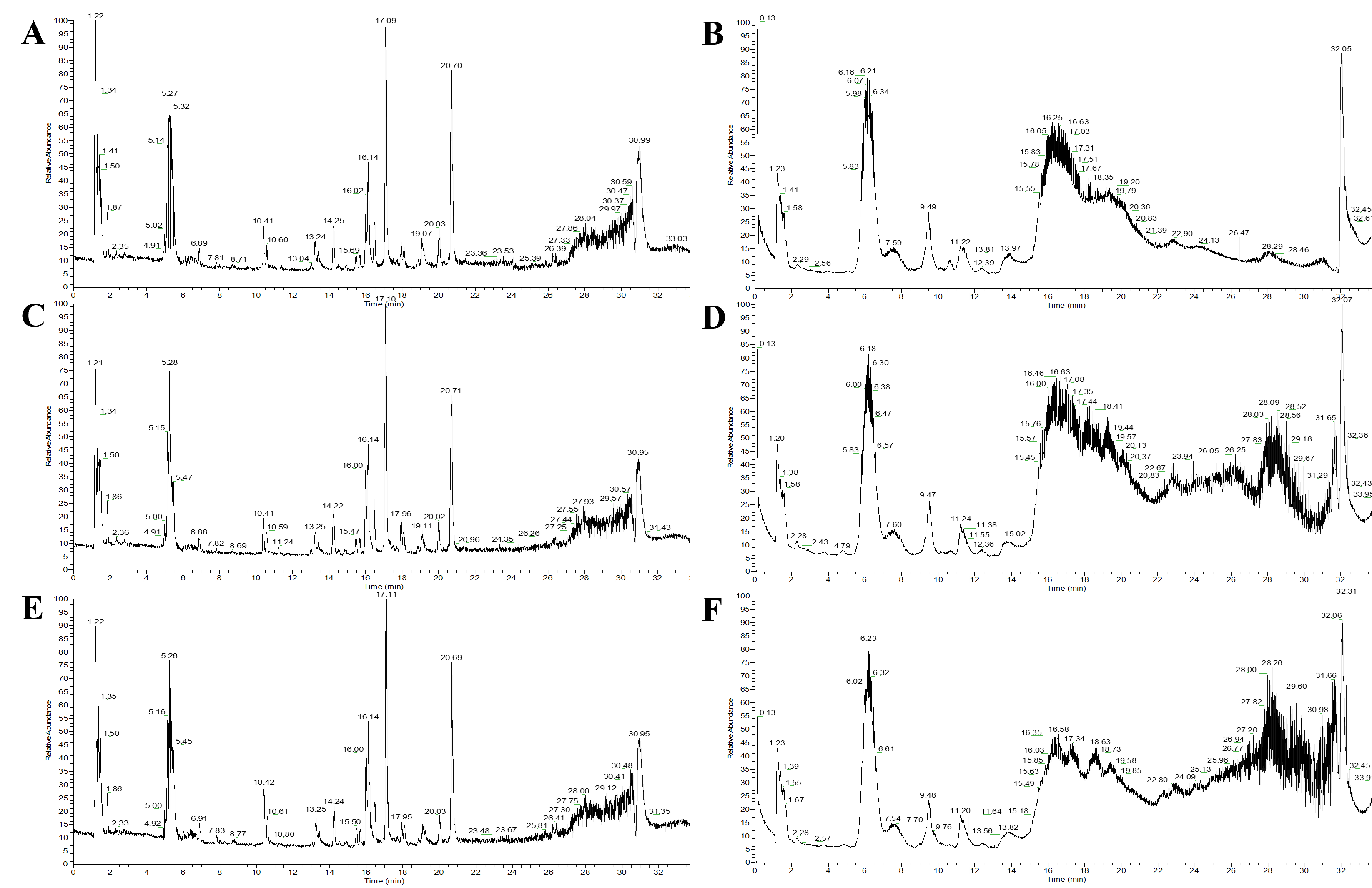

Supplement: Supplementary file 2 [file Image1.TIF]
