# Supplementary material for: Pharmacological activities and effective substances of the component-based Chinese medicine of Ginkgo biloba leaves based on serum pharmacochemistry, metabonomics and network pharmacology
Source: Front Pharmacol. 2023 Mar 10;14:1151447. doi: 10.3389/fphar.2023.1151447 (PMC10036596; doi:10.3389/fphar.2023.1151447)
Supplement: Supplementary file 3 [file DataSheet1.pdf]

Table S1 The detected ion chromatogram of constituents in GBCCM.

NO	T_R (min)	*m/z* Measured	*m/z* Calculated	δ/ppm	Identification	Formula	MS/MS Fragmentation	Classification
1	8.96	423.12973	423.12912	1.44	Ginkgolide J	$C_{20}H_{24}O_{10}$	423.12973; 379.14145	Terpene lactones
2	9.43	439.12436	439.12403	0.75	Ginkgolide C	$C_{20}H_{24}O_{11}$	439.12436; 383.13461	Terpene lactones
3	9.77	325.09329	325.09234	2.92	Bilobalide	$C_{15}H_{18}O_8$	325.09329; 251.09277	Terpene lactones
4	10.33	593.15155	593.15065	1.52	Kaempferol-3-O-rutinoside	$C_{27}H_{30}O_{15}$	593.15155; 285.04004	Flavonoid glycosides
5	11.51	447.09320	447.09273	1.05	Quercetin-3-O-rhamnoside	$C_{21}H_{20}O_{11}$	447.09320; 301.03452	Flavonoid glycosides
6	11.74	447.09332	447.09273	1.32	Kaempferol-3-O-glucoside	$C_{21}H_{20}O_{11}$	447.09332; 285.04028; 151.00275	Flavonoid glycosides
7	12.59	593.15125	593.15065	1.01	3-O-(β-D-glucosyl)-7-O-(α-L-rhamnosyl) kaempferol	$C_{27}H_{30}O_{15}$	593.15125; 285.03864	Flavonoid glycosides
8	12.97	287.05603	287.05556	1.64	Eriodictyol	$C_{15}H_{12}O_6$	287.05603;241.05133;177.05505;151.00259	Flavonoids
9	13.32	405.11893	405.11856	0.91	Ginkgolide K	$C_{20}H_{22}O_9$	405.11893	Terpene lactones
10	13.35	447.09201	447.09273	-1.51	Orientin	$C_{21}H_{20}O_{11}$	447.09201; 285.04025; 151.00301	Flavonoid glycosides
11	14.78	755.18115	755.18234	-1.58	Quercetin-3-O-[2-(6-p-coumaroyl-glucosyl)]-rhamnoside	$C_{36}H_{36}O_{18}$	755.18115; 301.03287	Flavonoid glycosides
12	17.27	407.13443	407.13420	0.56	Ginkgolide A	$C_{20}H_{24}O_9$	407.13443,363.14426, 351.14444,333.13361	Terpene lactones
13	17.30	423.13031	423.12912	2.81	Ginkgolide B	$C_{20}H_{24}O_{10}$	423.13031,367.13968	Terpene lactones
14	17.79	593.15021	593.15065	-0.74	Kaempferol-3-O-neohesperidoside	$C_{27}H_{30}O_{15}$	593.15021; 285.03928	Flavonoid glycosides
15	20.34	301.03519	301.03482	1.23	Quercetin	$C_{15}H_{10}O_7$	301.03519; 151.00264	Flavonoids
16	20.74	331.04572	331.04539	1.00	Laricitrin	$C_{16}H_{12}O_8$	331.04572; 151.00262	Flavonoids
17	23.12	739.18707	739.18743	-0.49	Kaempferol-3-O-2"-(6'"-p-coumaroyl) glucosylrhamnoside	$C_{36}H_{36}O_{17}$	739.18707; 285.03903	Flavonoid glycosides
18	29.60	285.04034	285.03991	1.51	Kaempferol	$C_{15}H_{10}O_6$	285.04034; 151.00276	Flavonoids
19	31.40	315.05075	315.05048	0.86	Isorhamnetin	$C_{16}H_{12}O_7$	315.05075; 151.00261; 285.02487	Flavonoids
